# Whole genomic approach in mutation discovery of infantile spasms patients

**DOI:** 10.3389/fneur.2022.944905

**Published:** 2022-07-22

**Authors:** Seungbok Lee, Sesong Jang, Jong-Il Kim, Jong Hee Chae, Ki Joong Kim, Byung Chan Lim

**Affiliations:** ^1^Department of Genomic Medicine, Seoul National University Hospital, Seoul, South Korea; ^2^Department of Pediatrics, Seoul National University College of Medicine, Seoul National University Children's Hospital, Seoul, South Korea; ^3^Department of Biomedical Sciences, Seoul National University College of Medicine, Seoul, South Korea; ^4^Medical Research Center, Genomic Medicine Institute (GMI), Seoul National University, Seoul, South Korea

**Keywords:** infantile spasms, whole-genome sequencing, epilepsy, genetic diagnosis, genomic medicine

## Abstract

Infantile spasms (IS) are a clinically and genetically heterogeneous group of epilepsy disorders in early infancy. The genetic backgrounds of IS have been gradually unraveled along with the increased application of next-generation sequencing (NGS). However, to date, only selected genomic regions have been sequenced using a targeted approach in most cases of IS, and the genetic etiologies of the majority of patients remain unknown. We conducted a proof-of-concept study using whole-genome sequencing (WGS) for the genetic diagnosis of IS. We included 16 patients with IS for this study, and WGS was applied as a first-tier test for genetic diagnosis. In total, we sequenced the whole genomes of 28 participants, including the genomes of six patients, which were sequenced with those of their parents. Among variants identified, we focused on those located in epilepsy or seizure-associated genes. We used two different methods to call relevant large deletions from WGS results. We found pathogenic or likely pathogenic variants in four patients (25.0%); a *de novo* variant in *HDAC4*, compound heterozygous variants in *GRM7*, and heterozygous variants in *CACNA1E* and *KMT2E*. We also selected two more candidate variants in *SOX5* and *SHROOM4* intronic regions. Although there are currently several difficulties in applying WGS for genetic diagnosis, especially in clinical interpretation of non-coding variants, we believe that developing sequencing technologies would overcome these hurdles in the near future. Considering the vast genetic heterogeneity and the substantial portion of patients with unknown etiologies, further studies using whole genomic approaches are necessary for patients with IS.

## Introduction

Infantile spasms (IS) are a clinically and genetically heterogeneous group of epileptic disorders characterized by clusters of brief contractions of the trunk and limb musculature in early infancy. Some 60–70% of patients with IS are shown to have associated medical conditions such as brain injuries, brain malformations, and chromosomal abnormalities (symptomatic IS), whereas disease etiologies of the others have been unclear in most cases (1).

Several genetic studies successfully identified causal mutations for IS in over 30 genes (2), and the clinical application of next-generation sequencing (NGS) has accelerated genetic diagnosis and novel gene discovery. There are currently almost one hundred genes associated with developmental and epileptic encephalopathy (DEE) in the Online Mendelian Inheritance in Man (OMIM) database (3). However, previous studies on IS genetics mostly relied on capture sequencing methods, including whole-exome sequencing or targeted panel sequencing, and to date, they can only explain <30% of cases (4, 5).

Whole-genome sequencing (WGS) is an advantageous method in genetic diagnosis due to the comprehensive coverage of all kinds of variants in the human genome, including copy number variation (CNV), translocation, and inversion. Several previous studies have demonstrated its application to early-onset epilepsy or DEE, and they proved a higher diagnostic efficacy for WGS of even up to nearly 70% when sequenced in a trio (6–9). However, many hurdles, such as cost, data processing, storage, and interpretation, have restricted the clinical application of WGS to only a minority of patients to date.

We are expecting the advance in NGS technologies gradually to resolve these burdens of WGS, by lowering the price and optimizing data handling. Likewise, in the field of genetics in epilepsy, WGS will play an increasing role in the near future. Here, we used the WGS method for patients with IS to demonstrate its clinical application.

## Materials and methods

### Study participants

In the present study, we included 16 patients with IS, and the genomes of six patients were sequenced with those of their parents. The patients had not undergone any genetic studies previously, and they were analyzed through WGS as a first-tier test. Inclusion criteria included patients with seizure onset before 12 months of age without structural abnormality on brain MRI. Pediatric neurologists diagnosed them as IS by their age of onset, seizure type, and electroencephalogram findings (Supplementary Table S1). We excluded infants who were suspected to have acquired etiologies such as hypoxic-ischemic encephalopathy, infection, inflammation, hemorrhage, and trauma. The newborn screening results for metabolic disorders were all negative in our patients. The study protocol was approved by the Institutional Review Board of Seoul National University Hospital (2007-192-1144), and the study was conducted in accordance with relevant guidelines and regulations.

### Whole-genome sequencing

The 28 samples including those from the 16 patients with IS were sequenced through WGS. Each sequencing library was prepared according to the manufacturer's instructions, which was paired-end sequenced on an Illumina HiSeq 2,500 system (Illumina, San Diego, CA, USA). Sequencing reads were aligned to the human reference genome (GRCh37) using the Burrows–Wheeler Aligner (10). We further processed the sequencing data following the Genome Analysis Toolkit (GATK) best-practice pipelines and called the sequence variants using the HaplotypeCaller in the GATK pipeline (11). The RefSeq gene database was used for the gene annotation.

### Sequence variation analysis

In the case of single-nucleotide variant (SNV) and short insertion/deletion (indel), a stepwise approach was used to narrow down pathogenic variants of our patients. (1) We first selected variants whose positions were genotyped in more than half of our study samples. (2) Variants located in repeat sequences or segmental duplications were also excluded using the RepeatMasker and genomicSuperDups tables of the University of California, Santa Cruz (UCSC) genome browser (http://genome.ucsc.edu) (12). (3) Next, we filtered the variants by allele frequency (AF) using the Genome Aggregation Database (gnomAD) and Kaviar (13, 14). The gnomAD version 2.1.1 was used for basic filtration, and the version 3.1 genome data were also used after alignment to GRCh38. Different levels of AF were applied for each variant; AF needs to be <0.001 for a recessive model and <0.00001 for a dominant model. (4) We initially focused on 165 curated epilepsy genes merging DEE genes from OMIM and epilepsy-related genes from ClinGen (15) and Epi25 (http://epi-25.org/). We next expanded to 1,553 seizure-associated genes, which were linked to the seizure phenotype in the Clinical Synopsis of OMIM, and 4,540 morbid OMIM genes.

In the case of trio samples, we screened *de novo* variants and homozygous or compound heterozygous variants for dominant and recessive models, respectively. By contrast, singleton cases required much more stringent filters in the selection of pathogenic variants as follows: (1) genotypes of variant positions called in >90% of cases, (2) biallelic sites allowing for one variant allele, (3) coverage depth ≥15 with variant allele fraction ≥0.3, (4) AF equal to zero in the gnomAD, Kaviar, KRGDB (16), TogoVar (https://togovar.biosciencedbc.jp/), and in-house databases, and (5) annotated to genes with dominant model diseases in the OMIM database. We also used some of these additional filters to select pathogenic variants in trio samples.

Non-silent variants, including non-synonymous SNVs, coding indels, and splicing variants, were further selected. In addition to the splicing variants on canonical splicing sites, our splicing variants also include those predicted by the dbscSNV program (RF_SCORE or ADA_SCORE above 0.6) and Combined Annotation-Dependent Depletion (CADD)-Splice (17, 18). Candidate variants were classified according to the international guidelines of the American College of Medical Genetics (ACMG) using InterVar (19, 20). We also used the CADD scores to predict the pathogenicity of SNVs (18) and screened the Human Gene Mutation Database (HGMD) and ClinVar databases to check whether candidate variants had been previously reported (21, 22).

### Structural variation analysis

We used Manta and Canvas, two distinct CNV callers, to call CNV events (23, 24), and trio samples were called together for each family when running the Manta program. We focused on large deletions among structural variations and selected relevant CNVs by filtering false calls according to the criteria suggested in a previous report, with some modifications (25). Common CNVs were defined as when their genomic regions were overlapped by ≥70%, and we narrowed down the common CNVs as follows: (1) not located in excluding regions including low mappability regions, centromeres, telomeres, segmental duplications, immunoglobulin, and human leukocyte antigen loci, (2) <50 Mb in length. CNVs called only in Manta need to meet the following additional criteria: (1) FILTER = ‘PASS,' (2) <1 Mb in length, (3) ‘IMPRECISE' not in INFO. In the case of CNVs called only in Canvas, we further removed CNVs with QUAL < 10 or called in outlier samples that were over-called compared with other samples (IS02, IS04, IS08, IS12, and IS16). We also checked whether our CNV candidates overlapped with morbid genes or pathogenic CNVs using the DECIPHER database (26).

## Results

### The whole genomic approach in variant analysis

The genome-wide coverage depth was as high as 62.0 × on average, and at least 94.8% of genomic positions were covered by ≥30 reads in every sample. The total number of sequence variants was 4.61 × 10^6^ on average (Supplementary Table S2).

**Figure 1:**
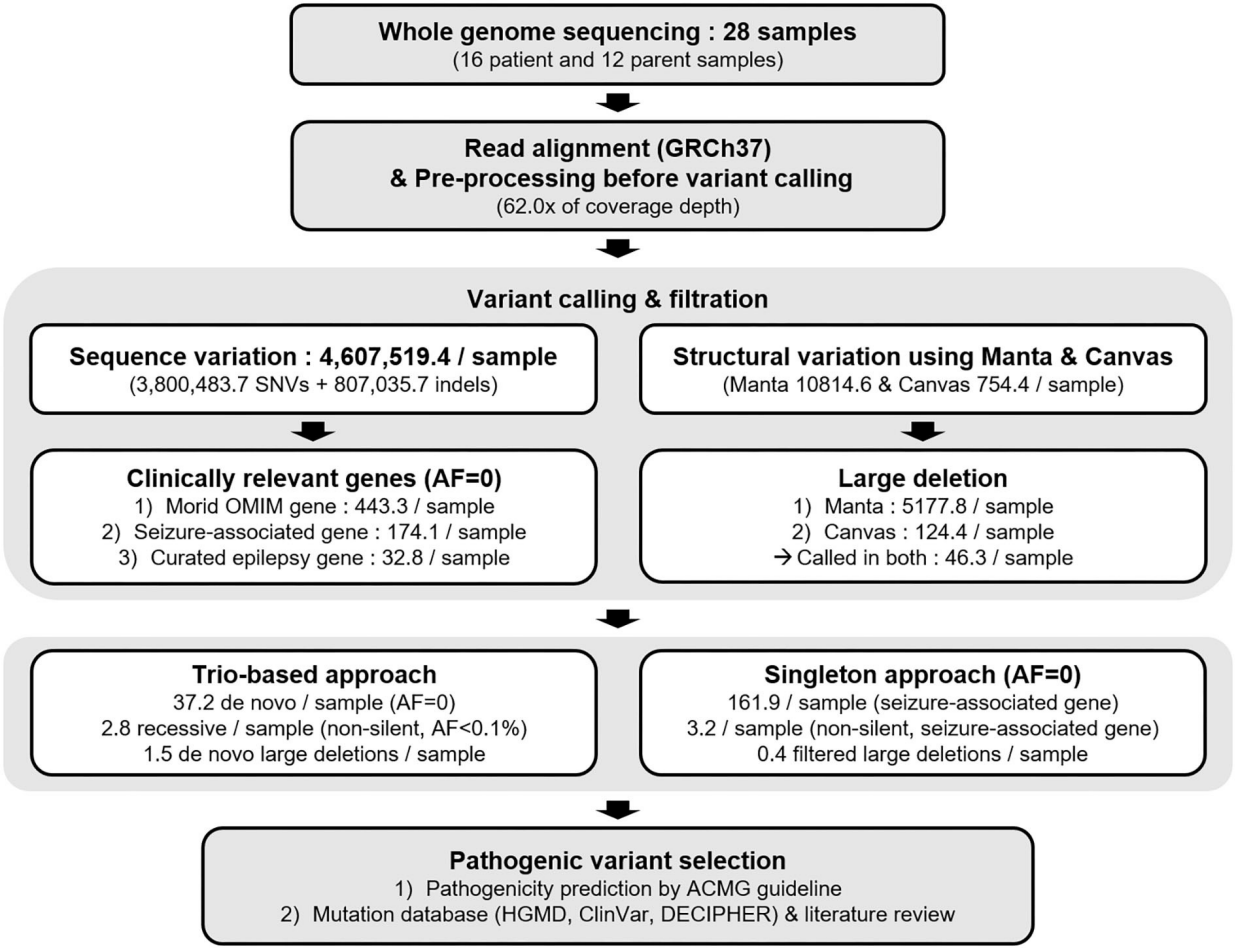
Overall scheme of whole-genome sequencing data analysis. This flowchart shows a schematic overview of the sequencing data analysis in the present study. In general, sequence and structural variants required independent approaches, from variant calling to pathogenic variant selection. In addition, trio samples were further evaluated for *de novo* and recessive variants in comparison with variants of their parents.

We simulated the filtration steps described in the Methods section (Figure 1). The initial filtration step, selecting variants genotyped in ≥50% of samples and not in repeat sequences or segmental duplications, could remove 58.4% of variants with 1.92 × 10^6^ variants per sample remaining. When the AF filters were set as 0.001 and 0.00001, only 2.58 and 2.08% among all variants were remaining, respectively. However, the actual count of variants after the filtration was still around one hundred thousand.

Next, we focused on the variants located in the clinically relevant gene; morbid OMIM genes, seizure-associated, and curated epilepsy genes. There were 6.78 × 10^3^ variants remaining for each sample when both filters of AF < 0.001 and morbid OMIM genes were applied, whereas the mean count of AF = 0 variants on curated epilepsy genes was 195.1 (Supplementary Figure S1).

### Mutation discovery in singleton samples

**Figure 2:**
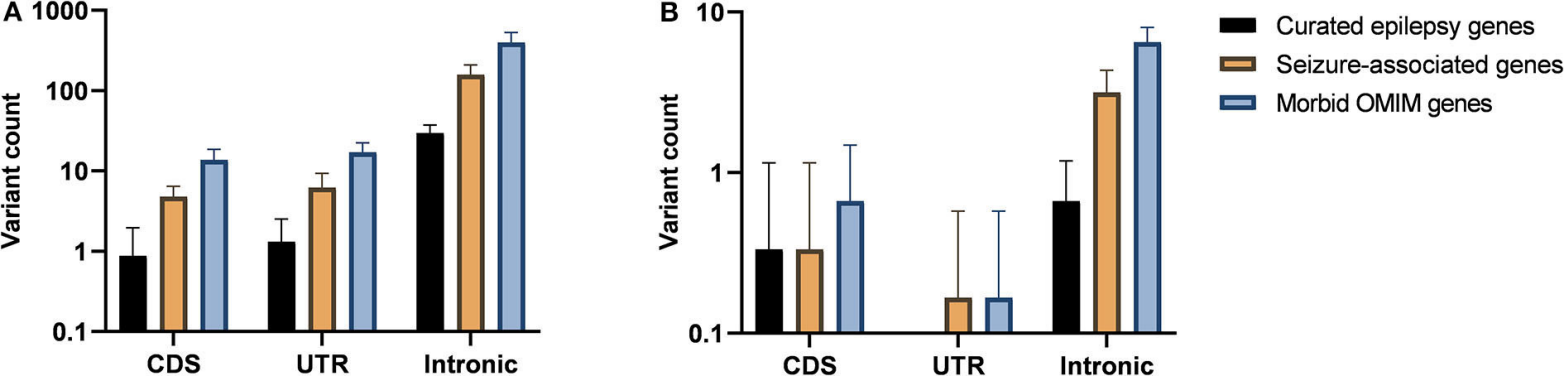
Sequence variant filtration by gene annotation. Mean variant counts are visualized in bar plots for curated epilepsy, seizure-associated, and morbid Online Mendelian Inheritance in Man (OMIM) genes. **(A)** Variants with allele frequencies = 0 were divided according to their gene annotation; coding sequence (CDS), untranslated region (UTR), and intron. **(B)** Among the variants visualized in **(A)**, we showed the numbers of *de novo* variants in the same way.

In addition to the filtration steps described, we attempted to find pathogenic variants in each patient (Table 1). Pathogenic variants for singleton cases were selected using all of the filters including AF equal to zero in multiple databases. When only variants on coding sequence were considered, there were around or <10 variants on curated epilepsy or seizure-associated genes for each sample (Figure 2A), and a total of 32 non-silent variants were remaining for a dominant model. According to the ACMG criteria, *CACNA1E* c.1807A>C (p.Ile603Leu) of IS11 was predicted to be likely pathogenic and *KMT2E* c.2632C>T (p.Gln878^*^) of IS15 was pathogenic. The *CACNA1E* variant was previously reported as a likely pathogenic variant in ClinVar. Although we also tried to detect variants for a recessive model in singleton samples using an AF filter <0.001, we could not select any pathogenic or likely pathogenic candidates due to a lack of parental genetic information.

**Table 1:** Pathogenic candidates discovered from whole-genome sequencing.

**Sample**	**Singleton /Trio**	**Gene**	**E/S/O**	**Gene ID**	**Nucleotide change**	**Amino-acid change**	**Variant type**	**Allele counts (ref/alt)**	**Inheritance**	**GnomAD (v2.1.1)**	**CADD score/ prediction**	**ClinVar reported**	**ACMG criteria**
**P/LP**													
IS01	Trio	*HDAC4*	*E*	NM_006037	c.2851A>T	p.Arg951*	De novo het	28 / 32	AD	not reported	42 / nonsense	no	P
IS02	Trio	*GRM7*	*S*	NM_000844	c.589C>T	p.Arg197Cys	Com het	36 / 33	AR	not reported	26.7 / missense	no	LP
					c.1972C>T	p.Arg658Trp	Com het	32 / 31		not reported	25.8 / missense	yes, P	LP
IS11	Singleton	*CACNA1E*	*E*	NM_000721	c.1807A>C	p.Ile603Leu	Het	38 / 34	AD	not reported	24.1 / missense	yes, P	LP
IS15	Singleton	*KMT2E*	*S*	NM_018682	c.2632C>T	p.Gln878*	Het	36 / 35	AD	not reported	39 / nonsense	no	P
**VUS**													
IS05	Trio	*SOX5*	*S*	NM_006940	c.38+11674C>T	NA	De novo het	29 / 28	AD	not reported	17.9 / intronic	no	VUS
IS06	Trio	*SHROOM4*	*S*	NM_020717	c.269+4A>G	NA	Hemi	0 / 38	XR	not reported	21.2 / splice site	no	VUS

E, curated epilepsy gene; S, seizure-associated gene; O, morbid OMIM gene; Het, heterozygous; Com het, compound heterozygous; Hemi, hemizygous; AD, autosomal dominant; AR, autosomal recessive; XR, X-linked recessive; P, pathogenic; LP, likely pathogenic; VUS, variant of unknown significance.

Supplementary Table S3 summarizes the counts of deletion calls for each sample. Manta called around 5,178 deletions on average for singleton cases. Canvas generated around 124 deletions for each sample, whereas five samples were over-called by more than three times compared with other samples (IS02, IS04, IS08, IS12, and IS16). Common deletions called in both programs were 46.3 for each sample, and further filters narrowed them down to only six deletions in total (Supplementary Table S4). However, none of them was predicted to be pathogenic or likely pathogenic; five were intergenic CNVs and one was located in exonic regions of *ITPR2* whose probability of being loss-of-function intolerant (pLI) score, calculated from gnomAD, is zero.

### Mutation discovery in trio samples

Using the parental genomic information and applying the four stringent filters as described in the Methods section (except the OMIM annotation), there were 223 *de novo* variants remaining in the six trios. Among these, the numbers of variants annotated to curated epilepsy, seizure-associated, and morbid OMIM genes were 6, 22, and 45, respectively (Figure 2B), and *HDAC4* c.2851A>T (p.Arg951^*^) in IS01 is the only non-silent variant in curated epilepsy or seizure-associated genes. This variant was predicted to be pathogenic by the ACMG criteria, and its CADD score was as high as 42.0.

Among the other 21 *de novo* variants in curated epilepsy or seizure-associated genes, two deep intronic variants on *SOX5* (IS05) and *WWOX* (IS06) had high CADD scores above 15 (17.92 and 17.91, respectively). Whereas, *SOX5* defects can cause Lamb–Shaffer syndrome (#616803) in an autosomal dominant manner, mutations in *WWOX* cause DEE in an autosomal recessive manner (#616211). Considering that only one allele was found in IS06, the *WWOX* variant is less like a pathogenic variant.

To discover recessive pathogenic variants, we selected non-silent variants and applied the three stringent filters, but not the OMIM annotation or AF filters. As a result, eight variants remained in five genes, all of which were found in the biallelic or hemizygous state in patients. Two genes among them were included in curated epilepsy genes, and we could find compound heterozygous variants of *GRM7*, c.589C>T (p.Arg197Cys), and c.1972C>T (p.Arg658Trp), and a hemizygous splicing variant of *SHROOM4* (c.269+4A>G). Both *GRM7* variants were predicted to be likely pathogenic according to ACMG guidelines, and one of them has been previously reported in ClinVar. In the case of the other variant of *SHROOM4*, although it is not located in canonical splicing sites, it was predicted to affect splicing in dbscSNV and CADD-Splice, and whose CADD score is as high as 21.2. However, it is still a variant of unknown significance (VUS) according to ACMG criteria because its pathogenicity remains as yet unclear.

Manta called approximately 7,736 deletions on average for trio cases. However, we could not find any pathogenic deletions in trio analysis.

## Discussion

The present study demonstrates the application of WGS to patients with IS using a tiered approach. We applied several different levels of filters in various aspects, including AF, clinically relevant gene sets (morbid OMIM genes, seizure-associated, and curated epilepsy genes), and gene annotations. To our knowledge, not many studies have conducted whole genomic approaches to IS to date. We discovered pathogenic or likely pathogenic variants in four patients (25.0%), including two trio samples (33.3% among trios), and two singleton samples (20.0% among singletons). In addition, we suggested candidate variants on seizure-associated genes in two more patients for whom pathogenic effects are as yet unclear. Therefore, we could suggest pathogenic or candidate variants in 37.5% of study participants using WGS. Considering that only six patients were sequenced with their parents in this study, the diagnostic rate seems similar to that of previous WGS studies (6–9).

In the process of data analysis, we selected variants on curated epilepsy or seizure-associated genes, which were derived from ClinGen, Epi25, and OMIM. Among the genes with *de novo* candidate variants, *HDAC4* has been clearly associated with seizure, including in patients with IS and DEE, in multiple studies since 2013 (5, 27, 28), and has been listed in Epi25 as an epilepsy-related gene. However, it took a long time for *HDAC4* to be enrolled in the OMIM database, and it has been recently linked to ‘neurodevelopmental disorder with central hypotonia and dysmorphic facies' (#619797) in OMIM during March 2022. In addition, there are also some curated epilepsy genes whose associations with seizures are not yet listed in the Clinical Synopsis of OMIM. Therefore, if we had filtered variants not in OMIM in the first step, some candidate variants might have been missed. Although the use of gene databases for variant filtration can be very useful to narrow down candidate variants, we need to be very careful in the choice of database and cautious about over-filtration.

We suggest the *SHROOM4* variant (c.269+4A>G) as one of the pathogenic candidates, although it is not located at canonical splicing sites. Two splicing prediction tools, dbscSNV and CADD-Splice, classified this as a splicing variant, and its CADD score is high at above 20. In particular, this variant is hemizygous and not reported in any kinds of variant databases we used (AF = 0). The *SHROOM4* gene is one of the curated epilepsy genes, and its pLI score is 1.0 suggesting that it would be intolerant of protein-truncating variants. Therefore, considering the splicing effect of variant and above gene information, we believe that it would be a highly probable pathogenic candidate for the patient (IS06).

As we can assume from the *SHROOM4* variant, the application of filters in the concept of functional prediction is not only crucial in pathogenic variant selection but also very subtle. Conventionally, as in this study, most researchers focus on non-silent variants changing amino acid sequences in proteins. However, this approach could miss synonymous exonic, deep intronic, and intergenic variants, which actually account for the majority of WGS calls. Although it is not easy and practicable at present, genome-wide screening of variants regardless of their gene annotations will soon become commonplace with the advancement of genomics.

The deep intronic variant of *SOX5* (c.38+11694C>T) in Table 1 is an example of a variant selected by such a genome-wide approach. *SOX5* mutations are known to cause Lamb–Shaffer syndrome (#616803) in an autosomal dominant manner, and the intronic variant of IS05 is a reliable *de novo* variant with high coverage depth (29× for reference allele and 28× for alternate allele). Although it remains a VUS because its pathogenic effect has not yet been validated, the *SOX5* variant could be a causal variant for IS05.

Despite the recent rapid growth of public variant databases, it is still insufficient to select clinically meaningful rare variants by AF filtration. Especially at the genome level, individual-specific rare variants, which are not reported in any variant databases (AF = 0), are nearly one hundred thousand. Although the gnomAD version 2.1.1 provides 125,748 exomes and 15,708 whole genomes and version 3.1 contains 76,156 whole genomes, only 780 and 2,604 East Asian genomes were included, respectively. Considering that most pathogenic variants are extremely rare, with an AF near zero, it is currently difficult to filter non-pathogenic and ethnic-specific polymorphisms with the public variant databases available.

Therefore, WGS is mostly insufficient with singleton analysis in genetic diagnosis, and important to use genomic and clinical information of other family members including parents (trio analysis), in consideration of their family history and inheritance patterns. However, in the present study, the number of *de novo* variants was approximately 35 on average and further filtration was eventually required, such as the use of gene and repeat sequence annotation, or functional predictions derived from various tools. Chiefly, literature reviews and screening of mutation databases, including ClinVar and HGMD, are essential processes and provide the strongest evidence in the selection of pathogenic variants.

We could not find any pathogenic structural variations in the study patients. Because we used WGS as a first-tier test for genetic diagnosis and the proportion of structural variants in DEE has been estimated to be near to or <10% (8, 9), there might be no true pathogenic structural variants in our 16 study participants. Moreover, the interpretation of structural variant calls is more complex than that for sequence variants; different programs or options can generate different CNV calls (Supplementary Table S2), and there is a paucity of public databases for variant filtration compared with those for sequence variation. Nevertheless, advances in CNV calling and filtering strategies will overcome such hurdles gradually and CNV analysis will soon become standardized and pervasive (29).

## Conclusion

We demonstrated the utility of WGS in patients with IS and tried to maximize the advantage of WGS using various kinds of approaches, such as non-coding and structural variant analysis, trio-based analysis, phenotype-oriented approaches, and utilization of multiple variant databases and prediction tools. However, current approaches to determine pathogenicity have been mostly established for coding variants only, and pathogenicity or biological effect prediction for non-coding variants remains quite challenging. Although we could suggest two intronic variants by trio-based and phenotype-oriented approaches, the diagnostic yield of WGS as a first-tier test in IS in this study is similar to that of whole-exome sequencing or targeted panel sequencing (4, 5). Currently, no single pipeline, database, or program is superior to the others and solely sufficient for pathogenic variant detection, and multiple approaches need to be complementary. Meanwhile, we should accumulate much more genomic variant information, especially by ethnicity, and develop more accurate functional prediction tools for non-coding variants.

## Data availability statement

The datasets presented in this study can be found in online repositories. The name of the repository and accession number can be found below: National Center for Biotechenology Information (NCBI) BioProject, https://www.ncbi.nlm.nih.gov/bioproject/, PRJNA850643.

## Ethics statement

The studies involving human participants were reviewed and approved by the Institutional Review Board of Seoul National University Hospital. Written informed consent to participate in this study was provided by the participants' legal guardian/next of kin.

## Author contributions

Study conception and design: JC, KK, and BL. Data collection, results interpretation, and drafting the manuscript: SL and BL. Data analysis: SL, SJ, and J-IK. Manuscript revision and approval: All authors.

## Funding

This study was supported by grant no. 0320200350 from the Seoul National University Hospital Research Fund.

## Conflict of interest

The authors declare that the research was conducted in the absence of any commercial or financial relationships that could be construed as a potential conflict of interest.

## Publisher's note

All claims expressed in this article are solely those of the authors and do not necessarily represent those of their affiliated organizations, or those of the publisher, the editors and the reviewers. Any product that may be evaluated in this article, or claim that may be made by its manufacturer, is not guaranteed or endorsed by the publisher.
