# Supplementary material for: Whole genomic approach in mutation discovery of infantile spasms patients
Source: Front Neurol. 2022 Jul 22;13:944905. doi: 10.3389/fneur.2022.944905 (PMC9354570; doi:10.3389/fneur.2022.944905)
Supplement: Supplementary file 1 [file Image_1.PDF]

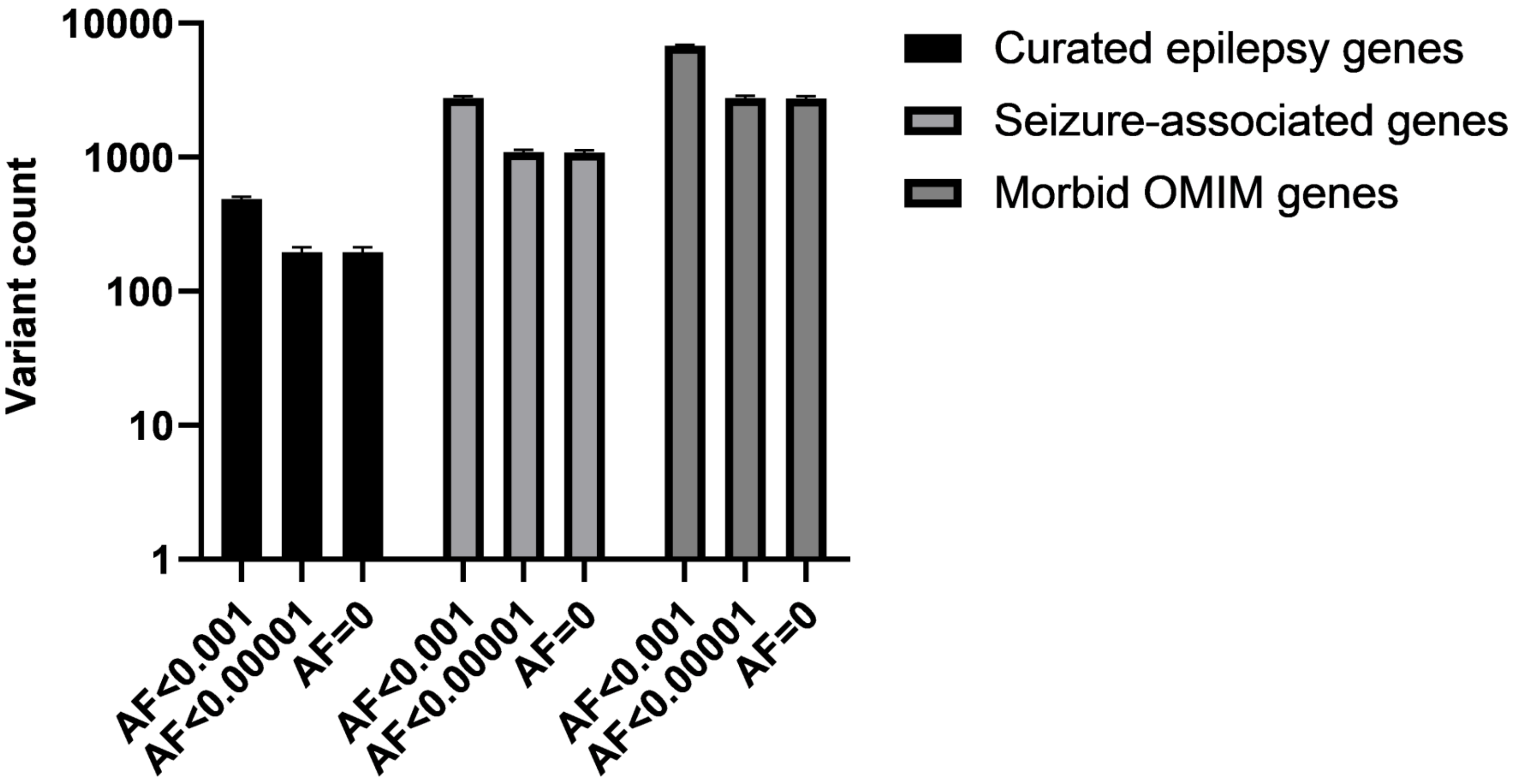

Supplementary Figure S1 . Sequence variant filtration by allele frequency. Mean variant counts were visualized in bar plots when different filters were used. We applied allele three different levels of frequency (AF) filtrations to variants on curated epilepsy, seizure-associated, and morbid OMIM genes, respectively.
